# Supplementary material for: Changes of Ex Vivo Cervical Epithelial Cells Due to Electroporation with JMY
Source: Int J Mol Sci. 2023 Nov 28;24(23):16863. doi: 10.3390/ijms242316863 (PMC10706833; doi:10.3390/ijms242316863)
Supplement: Supplementary file 1 [file ijms-24-16863-s001.zip › ijms-2680010-supplementary.pdf]

One Way ANOVA of cell number								
	N Analysis	N Missing	Mean	Standard Deviation	SE of Mean			
Nc	240	28	47	22.3618	1.44345			
Nc JMY	218	20	31.96774	17.30458	1.09884			
Ic	104	164	30.38462	10.25719	1.0058			
Ic JMY	99	130	16.37681	9.60627	0.81774			
Nc elec	120	148	61.73333	24.46254	2.23311			
At the 0.05 level, the population means are significantly different.								
Overall								
	DF	Sum of Squares	Mean Square	F Value	Prob>F			
Model	4	166529.7749	41632.44373	122.08028	0			
Error	845	288166.2298	341.02512					
Total	849	454696.0047						
Fit Statistic								
	R-Square	Coeff Var	Root MSE	Data Mean				
	0.36624	0.48997	18.46687	37.68941				
Bonferroni test								
	MeanDiff	SEM	t Value	Prob	Alpha	Sig	LCL	UCL
Nc vs. Nc JMY	-15.03226	1.67214	-8.98985	1.59E-17	0.05	1	-19.73836	-10.32615
Nc vs. Ic	-16.61538	2.16795	-7.66408	4.94E-13	0.05	1	-22.71693	-10.51384
Nc JMY vs. Ic	-1.58313	2.15736	-0.73383	1	0.05	0	-7.65485	4.4886
Ic JMY vs. Nc	-30.62319	1.97285	-15.52231	5.40E-47	0.05	1	-36.17563	-25.07075
Ic JMY vs. Nc JMY	-15.59093	1.9612	-7.9497	5.97E-14	0.05	1	-21.11058	-10.07128
Ic JMY vs. Ic	-14.0078	2.39798	-5.84151	7.38E-08	0.05	1	-20.75673	-7.25888
Nc elec vs. Nc	14.73333	2.06466	7.13597	2.07E-11	0.05	1	8.92251	20.54416
Nc elec vs. Nc JMY	29.76559	2.05353	14.49486	1.10E-41	0.05	1	23.98609	35.54509
Nc elec vs. Ic	31.34872	2.47406	12.67097	8.22E-33	0.05	1	24.38566	38.31177
Nc elec vs. Ic JMY	45.35652	2.30501	19.67736	2.97E-70	0.05	1	38.86924	51.8438

Supplement Table S1. Results of ANOVA and Bonferroni test on data of cell number in different cases. The variance of average number of cells in a unit area in different cases data from Figure 1E were tested with ANOVA in Origin 2018. At the 0.05 level, the means of the populations are significantly different. Based on their variance, the Bonferroni test shows that only the comparison of $Nc_{elect.+JMY}$ vs. Ic resulted in no significant differences. (Sig. level 0 means NOT significant, level 1 means a significant difference)

One Way ANOVA of cell number								
	N Analysis	N Missing	Mean	tandard Deviatio	SE of Mean			
pre	80	172	52.67089	27.22448	3.06299			
pre JMY	80	172	43.27848	16.36047	1.8407			
post	160	91	44.35	19.01016	1.50289			
post JMY	168	83	26.71429	15.12865	1.1672			
At the 0.05 level, the population means are significantly different.								
Overall								
	DF	Sum of Squares	Mean Square	F Value	Prob>F			
Model	3	46057.25709	15352.41903	42.43724	0			
Error	482	174372.0022	361.76764					
Total	485	220429.2593						
Fit Statistic								
	R-Square	Coeff Var	Root MSE	Data Mean				
	0.20894	0.48235	19.02019	39.4321				
Bonferroni test								
	MeanDiff	SEM	t Value	Prob	Alpha	Sig	LCL	UCL
pre vs. pre JMY	-9.39241	3.02633	-3.10356	1.22E-02	0.05	1	-17.40974	-1.37507
post vs. pre	-8.32089	2.61541	-3.18148	9.36E-03	0.05	1	-15.24962	-1.39215
post vs. pre JMY	1.07152	2.61541	0.40969	1	0.05	0	-5.85721	8.00025
post JMY vs. pre	-25.9566	2.59475	-10.00352	9.37E-21	0.05	1	-32.83059	-19.08261
ost JMY vs. pre JM	-16.5642	2.59475	-6.38374	2.44E-09	0.05	1	-23.43818	-9.69021
post vs. post JMY	-17.63571	2.10105	-8.39375	3.17E-15	0.05	1	-23.20181	-12.06962

Supplement Table S2. Results of ANOVA and Bonferroni test on data of cell number in post-menopausal and pre-menopausal cases. The variance of the average number of cells in a unit area in pre-menopausal and post-menopausal cases data from Figure 1F were tested with ANOVA in Origin 2018. At the 0.05 level, the means of the populations are significantly different. Based on their variance, the Bonferroni test shows that only the comparison of $Nc_{pre.elect.+JMY}$ vs. $Nc_{post.}$ resulted in no significant differences. (Sig. level 0 means NOT significant, level 1 means a significant difference)

One Way ANOVA of cell size								
	N Analysis	N Missing	Mean	Standard Deviation	SE of Mean			
Nc	645	88	50.35437	9.73427	0.38329			
Nc JMY	463	270	50.51439	9.00816	0.41864			
Ic	108	625	47.89408	10.78269	1.03757			
Ic JMY	138	623	44.0269	8.43852	0.80458			
Nc elec	99	657	53.00833	8.73022	1.00142			
At the 0.05 level, the population means are significantly different.								
Overall								
	DF	Sum of Squares	Mean Square	F Value	Prob>F			
Model	4	5269.33918	1317.3348	14.78983	7.70E-12			
Error	1397	124431.2228	89.07031					
Total	1453	129700.562						
Fit Statistic								
	R-Square	Coeff Var	Root MSE	Data Mean				
	0.04063	0.18926	9.43771	49.86511				
Bonferroni test								
	MeanDiff	SEM	t Value	Prob	Alpha	Sig	LCL	UCL
Nc vs. Nc JMY	0.16003	0.57487	0.27837	1.00E+00	0.05	0	-1.45621	1.77626
Nc vs. Ic	-2.46028	0.98123	-2.50734	1.23E-01	0.05	0	-5.21902	0.29845
Nc JMY vs. Ic	-2.62031	1.00851	-2.59819	0.0947	0.05	0	-5.45575	0.21513
Ic JMY vs. Nc	-6.32747	0.97356	-6.49929	1.12E-09	0.05	1	-9.06464	-3.5903
Ic JMY vs. Nc JMY	-6.48749	1.00105	-6.48067	1.26E-09	0.05	1	-9.30195	-3.67303
Ic JMY vs. Ic	-3.86718	1.27846	-3.02488	2.53E-02	0.05	1	-7.46157	-0.2728
Nc elec vs. Nc	2.65396	1.14458	2.31871	2.06E-01	0.05	0	-0.56404	5.87196
Nc elec vs. Nc JMY	2.49394	1.16806	2.13512	3.29E-01	0.05	0	-0.79005	5.77793
Nc elec vs. Ic	5.11425	1.41305	3.6193	3.06E-03	0.05	1	1.14146	9.08703
Nc elec vs. Ic JMY	8.98143	1.40773	6.38007	2.40E-09	0.05	1	5.02359	12.93927

Supplement Table S3. Results of ANOVA and Bonferroni test on data of cell size in different cases. The variance of cell size in different cases data from Figure 4A were tested with ANOVA in Origin 2018. At the 0.05 level, the means of the populations are significantly different. Based on their variance, the Bonferroni test shows that only the comparisons of $Ic_{elect.+JMY}$ vs. Nc; $Ic_{elect.+JMY}$ vs. $Nc_{elect.+JMY}$; $Ic_{elect.+JMY}$ vs. Ic; $Nc_{elect.}$ vs. Ic; $Nc_{elect.}$ vs. $Ic_{elect.+JMY}$; resulted in significant differences. (Sig. level 0 means NOT significant, level 1 means a significant difference)

One Way ANOVA of nuclear size								
	N Analysis	N Missing	Mean	Standard Deviation	SE of Mean			
Nc	900	64	8.7033	1.97117	0.06571			
Nc JMY	577	387	7.29156	1.64135	0.06833			
Ic	138	826	8.52838	1.4591	0.12421			
Ic JMY	143	821	9.40149	1.84759	0.1545			
Nc elec	99	865	8.86038	1.06183	0.10672			
At the 0.05 level, the population means are significantly different.								
Overall								
	DF	Sum of Squares	Mean Square	F Value	Prob>F			
Model	4	944.59667	236.14917	73.73017	0.00E+00			
Error	1852	5931.74094	3.20288					
Total	1856	6876.33761						
Fit Statistic								
	R-Square	Coeff Var	Root MSE	Data Mean				
	0.13737	0.21526	1.78966	8.31379				
Bonferroni test								
	MeanDiff	SEM	t Value	Prob	Alpha	Sig	LCL	UCL
Nc vs. Nc JMY	-1.41174	0.09544	-14.79115	7.08E-46	0.05	1	-1.67997	-1.1435
Nc vs. Ic	-0.17491	0.16361	-1.06909	1.00E+00	0.05	0	-0.63472	0.2849
Nc JMY vs. Ic	1.23682	0.16959	7.29309	4.47E-12	0.05	1	0.76021	1.71344
Ic JMY vs. Nc	0.69819	0.16111	4.33363	1.55E-04	0.05	1	0.24541	1.15098
Ic JMY vs. Nc JMY	2.10993	0.16718	12.6208	4.32E-34	0.05	1	1.64009	2.57977
Ic JMY vs. Ic	0.87311	0.21356	4.08837	4.53E-04	0.05	1	0.27292	1.47329
Nc elec vs. Nc	0.15709	0.1895	0.82895	1.00E+00	0.05	0	-0.37549	0.68966
Nc elec vs. Nc JMY	1.56882	0.19469	8.05816	1.37E-14	0.05	1	1.02167	2.11597
Nc elec vs. Ic	0.332	0.23572	1.40848	1.00E+00	0.05	0	-0.33045	0.99445
Nc elec vs. Ic JMY	-0.54111	0.23399	-2.31254	2.09E-01	0.05	0	-1.1987	0.11649

Supplement Table S4. Results of ANOVA and Bonferroni test on data of nuclear size in different cases. The variance of nuclear size in different cases data from Figure 4B were tested with ANOVA in Origin 2018. At the 0.05 level, the means of the populations are significantly different. Based on their variance, the Bonferroni test shows that only the comparisons of Ic vs. Nc; Nc vs. $Nc_{elect.}$; $Nc_{elect.}$ vs. Ic; $Nc_{elect.}$ vs. $Ic_{elect.+JMY}$; resulted in no significant differences. (Sig. level 0 means NOT significant, level 1 means a significant difference)

**One Way ANOVA nuclear/cell size ratio**								
	**N Analysis**	**N Missing**	**Mean**	**Standard Deviation**	**SE of Mean**			
**Nc**	624	29	0.17374	0.04863	0.00195			
**Nc JMY**	463	190	0.14904	0.04499	0.00209			
**Ic**	104	549	0.18154	0.04403	0.00432			
**Ic JMY**	105	548	0.21	0.04699	0.00459			
**Nc elec**	74	579	0.17025	0.03243	0.00377			
**At the 0.05 level, the population means are significantly different.**								
**Overall**								
	**DF**	**Sum of Squares**	**Mean Square**	**F Value**	**Prob>F**			
**Model**	4	0.39113	0.09778	45.79592	0.00E+00			
**Error**	1365	2.91452	0.00214					
**Total**	1369	3.30565						
**Fit Statistic**								
	**R-Square**	**Coeff Var**	**Root MSE**	**Data Mean**				
	0.11832	0.27411	0.04621	0.16858				
**Bonferroni test**								
	**MeanDiff**	**SEM**	**t Value**	**Prob**	**Alpha**	**Sig**	**LCL**	**UCL**
**Nc vs. Nc JMY**	-0.0247	0.00283	-8.71369	8.37E-17	0.05	1	-0.03267	-0.01673
**Nc vs. Ic**	0.0078	0.00489	1.59435	1.00E+00	0.05	0	-0.00596	0.02156
**Nc JMY vs. Ic**	0.0325	0.00501	6.48165	1.26E-09	0.05	1	0.0184	0.0466
**Ic JMY vs. Nc**	0.03626	0.00487	7.43843	1.79E-12	0.05	1	0.02255	0.04996
**Ic JMY vs. Nc JMY**	0.06095	0.00499	12.20363	1.38E-31	0.05	1	0.04691	0.075
**Ic JMY vs. Ic**	0.02845	0.00639	4.45087	9.25E-05	0.05	1	0.01048	0.04643
**Nc elec vs. Nc**	-0.00349	0.00568	-0.6141	1.00E+00	0.05	0	-0.01946	0.01248
**Nc elec vs. Nc JMY**	0.02121	0.00578	3.66618	2.56E-03	0.05	1	0.00494	0.03747
**Nc elec vs. Ic**	-0.01129	0.00703	-1.60681	1.00E+00	0.05	0	-0.03105	0.00847
**Nc elec vs. Ic JMY**	-0.03974	0.00701	-5.66687	1.77E-07	0.05	1	-0.05946	-0.02003

Supplement Table S5. Results of ANOVA and Bonferroni test on data of nuclear/cell size ratio in different cases. The variance of nuclear/cell size ratio in different cases data from Figure 4C were tested with ANOVA in Origin 2018. At the 0.05 level, the means of the populations are significantly different. Based on their variance, the Bonferroni test shows that only the comparisons of Ic vs. Nc; Nc vs. $Nc_{elect.}$; $Nc_{elect.}$ vs. Ic; resulted no significant differences. (Sig. level 0 means NOT significant, level 1 means a significant difference)

One Way ANOVA of cell size								
	**N Analysis**	**N Missing**	**Mean**	**tandard Deviatior**	**SE of Mean**			
**pre**	130	253	50.42838	10.17907	0.89276			
**pre JMY**	122	261	50.69058	8.98589	0.81354			
**post**	382	1	50.09606	10.47902	0.53615			
**post JMY**	339	44	50.44688	9.04746	0.49139			
**At the 0.05 level, the population means are NOT significantly different.**								
**Overall**								
	**DF**	**Sum of Squares**	**Mean Square**	**F Value**	**Prob>F**			
**Model**	3	42.60658	14.20219	0.14855	0.93063			
**Error**	969	92641.52731	95.60529					
**Total**	972	92684.13389						
**Fit Statistic**								
	**R-Square**	**Coeff Var**	**Root MSE**	**Data Mean**				
	4.60E-04	0.19425	9.7778	50.33724				
**Bonferroni test**								
	**MeanDiff**	**SEM**	**t Value**	**Prob**	**Alpha**	**Sig**	**LCL**	**UCL**
**pre vs. pre JMY**	0.26221	1.23251	0.21274	1.00E+00	0.05	0	-2.99616	3.52057
**post vs. pre**	-0.33231	0.99282	-0.33472	1.00E+00	0.05	0	-2.95703	2.2924
**post vs. pre JMY**	-0.59452	1.01682	-0.58468	1	0.05	0	-3.28268	2.09364
**post JMY vs. pre**	0.01851	1.00869	0.01835	1.00E+00	0.05	0	-2.64814	2.68516
**ost JMY vs. pre JM**	-0.2437	1.03231	-0.23607	1.00E+00	0.05	0	-2.97281	2.48542
**post vs. post JMY**	0.35082	0.72959	0.48085	1.00E+00	0.05	0	-1.57798	2.27962

Supplement Table S6. Results of ANOVA and Bonferroni test on data of cell size in post-menopausal and pre-menopausal cases. The variance of cell size in pre-menopausal and post-menopausal cases data from Figure 5A were tested with ANOVA in Origin 2018. At the 0.05 level, the means of the populations are not significantly different. Based on their variance, the Bonferroni test shows that the comparisons resulted in no significant differences. (Sig. level 0 means NOT significant, level 1 means a significant difference)

One Way ANOVA of nuclear size								
	N Analysis	N Missing	Mean	Standard Deviation	SE of Mean			
pre	148	431	7.47999	1.63403	0.13432			
pre JMY	184	395	7.01784	1.57683	0.11625			
post	573	6	9.20678	2.05778	0.08597			
post JMY	390	189	7.40087	1.61511	0.08178			
At the 0.05 level, the population means are significantly different.								
Overall								
	DF	Sum of Squares	Mean Square	F Value	Prob>F			
Model	3	1161.03965	387.01322	116.61808	0			
Error	1291	4284.36189	3.31864					
Total	1294	5445.40154						
Fit Statistic								
	R-Square	Coeff Var	Root MSE	Data Mean				
	2.13E-01	0.2234	1.82171	8.15455				
Bonferroni test								
	MeanDiff	SEM	t Value	Prob	Alpha	Sig	LCL	UCL
pre vs. pre JMY	-0.46215	0.20114	-2.2976	1.30E-01	0.05	0	-0.99364	0.06934
post vs. pre	1.72679	0.16797	10.28014	4.20E-23	0.05	1	1.28295	2.17063
post vs. pre JMY	2.18894	0.15436	14.1805	9.47E-42	0.05	1	1.78106	2.59681
post JMY vs. pre	-0.07912	0.17588	-0.44986	1.00E+00	0.05	0	-0.54384	0.3856
ost JMY vs. pre JM	0.38303	0.16293	2.35092	1.13E-01	0.05	0	-0.04748	0.81354
post vs. post JMY	-1.80591	0.11959	-15.10121	8.57E-47	0.05	1	-2.12189	-1.48992

Supplement Table S7. Results of ANOVA and Bonferroni test on data of nuclear size in post-menopausal and pre-menopausal cases. The variance of nuclear size in pre-menopausal and post-menopausal cases data from Figure 5B were tested with ANOVA in Origin 2018. At the 0.05 level, the means of the populations are significantly different. Based on their variance, the Bonferroni test shows that only the comparisons of $Nc_{pre.}$ vs. $Nc_{pre.elect.+JMY}$; $Nc_{pre.}$ vs. $Nc_{post.elect.+JMY}$; $Nc_{post.elect.+JMY}$ vs. $Nc_{pre.elect.+JMY}$; resulted in no significant differences. (Sig. level 0 means NOT significant, level 1 means a significant difference)

One Way ANOVA of nuclear/cell size ratio								
	N Analysis	N Missing	Mean	tandard Deviatior	SE of Mean			
pre	131	266	0.15399	0.04606	0.00402			
pre JMY	122	275	0.14173	0.04411	0.00399			
post	356	41	0.18341	0.04862	0.00258			
post JMY	340	57	0.1515	0.04305	0.00233			
At the 0.05 level, the population means are significantly different.								
Overall								
	DF	Sum of Squares	Mean Square	F Value	Prob>F			
Model	3	0.25896	0.08632	41.22289	0			
Error	945	1.9788	0.00209					
Total	948	2.23776						
Fit Statistic								
	R-Square	Coeff Var	Root MSE	Data Mean				
	1.16E-01	0.2815	0.04576	0.16256				
Bonferroni test								
	MeanDiff	SEM	t Value	Prob	Alpha	Sig	LCL	UCL
pre vs. pre JMY	-0.01226	0.00576	-2.12983	2.01E-01	0.05	0	-0.02748	0.00296
post vs. pre	0.02943	0.00468	6.29257	2.86E-09	0.05	1	0.01706	0.04179
post vs. pre JMY	0.04169	0.0048	8.68382	9.96E-17	0.05	1	0.029	0.05438
post JMY vs. pre	-0.00249	0.00471	-0.52816	1.00E+00	0.05	0	-0.01493	0.00996
ost JMY vs. pre JM	0.00978	0.00483	2.02452	2.59E-01	0.05	0	-0.00299	0.02254
post vs. post JMY	-0.03191	0.00347	-9.19615	1.39E-18	0.05	1	-0.04108	-0.02274

Supplement Table S8. Results of ANOVA and Bonferroni test on data of nuclear/cell size ratio in post-menopausal and pre-menopausal cases. The variance of nuclear/cell size ratio in pre-menopausal and post-menopausal cases data from Figure 5C were tested with ANOVA in Origin 2018. At the 0.05 level, the means of the populations are significantly different. Based on their variance, the Bonferroni test shows that only the comparisons of $Nc_{pre.}$ vs. $Nc_{pre.elect.+JMY}$; $Nc_{pre.}$ vs. $Nc_{post.elect.+JMY}$; $Nc_{post.elect.+JMY}$ vs. $Nc_{pre.elect.+JMY}$; resulted no significant differences. (Sig. level 0 means NOT significant, level 1 means a significant difference)
